# Potent antitumor property of *Allium bakhtiaricum* extracts

**DOI:** 10.1186/s12906-019-2522-8

**Published:** 2019-06-04

**Authors:** Kosar Vafaee, Soudeh Dehghani, Raheleh Tahmasvand, Farzaneh Saeed Abadi, Saeed Irian, Mona Salimi

**Affiliations:** 1 Department of Cell and Molecular Biology, Faculty of Biological Sciences, Kharazmi University, P.O. Box 15719-14911, Tehran, Iran; 2 Department of Physiology and Pharmacology, Pasteur Institute of Iran, P.O. Box 1316943551, Tehran, Iran

**Keywords:** *Allium bakhtiaricum*, Breast cancer, Fraction, Balb/c mice, Cell cycle

## Abstract

**Background:**

Allium species are magnificently nutritious and are commonly used as a part of the diet in Iran. They have health enhancing benefits including anticancer properties due to the presence of numerous bioactive compounds. Herein, we investigated in vitro and in vivo anticancer properties of *Allium bakhtiaricum* extracts.

**Methods:**

Anti-growth activity of different fractions was explored in vitro on different cancerous cells using MTT assay, Annexin V/PI and SA-β-gal staining, Western blotting, flowcytometric and immunofluorescence microscopic evaluations. In vivo antitumor activity was investigated in BALB/c mice bearing 4 T1 mammary carcinoma cells.

**Results:**

We demonstrated that chloroformic and ethyl acetate fractions exert cytotoxic activity toward MDA-MB-231 cells, the most sensitive cell line, after 72 h of treatment with IC_50_ values of 0.005 and 0.006 mg/ml, respectively. Incubation of MDA-MB-231 cells with ¼ and ½ IC_50-72h_ concentrations of each fraction resulted in a significant G2/M cell cycle arrest. ¼ IC_50-72h_ concentration of the chloroform fraction led to the disruption of polymerization in mitotic microtubules. Exposure of human breast cancer cells to different concentrations of the extracts at different incubation times did not induce apoptosis, autophagy or senescence. Our in vivo study revealed that administration of the chloroform extract at a dose of 1 mg/kg/day strongly suppressed mammary tumor progression and decreased the number of proliferative cells in the lung tissues indicating its anti-metastatic effect.

**Conclusion:**

Our findings imply that the chloroform fraction of *Allium bakhtiaricum* possesses the suppressive action on breast cancer through mitotic cell cycle arrest suggesting a mechanism associated with disturbing microtubule polymerization.

**Electronic supplementary material:**

The online version of this article (10.1186/s12906-019-2522-8) contains supplementary material, which is available to authorized users.

## Background

Cancer is considered to be a noticeable disease with worldwide distribution distinguished by uncontrolled growth and spreading the abnormal cells, which currently causes million deaths. Based on the World Health Organization (WHO) report, around 15 million new cases of cancer is estimated by 2020 [1, 2]. In particular, 60% of the world population is accounted for Asia which includes half the total burden of cancer [3]. In this regard, among Asian countries, about 50,800 new cancer cases take place in Iran, annually [4].

Cancer treatment generally consists of surgery, chemotherapy, radiotherapy, or a mixture of them, among which chemotherapy is the most effective approach to control the disease. However, chemotherapy causes adverse and inevitable side effects for the patients [5], which currently limits its application. To address this issue, using herbal medicine has gained a great attention. Plants are an invaluable source of natural products, including phytochemicals, commonly known as secondary metabolites, with diverse therapeutic applications [6–8]. Recently, numerous studies have shown that the use of natural products for cancer treatment results in fewer or diminished side effects as well as a longer survival period for patients [9, 10].

Importantly, folk medicine has strong historical and cultural roots in Iran, which dates back to the Old Babylonian and Assyrian periods [11, 12]. Traditional remedy has been long used in the treatment of a great number of diseases in Iran [13]. Allium is the largest and generally most important genus in the Alliaceae family and has been used as folk medicine [14, 15]. Of note, Iran is one of the valuable resources of Allium including 121 wild Allium species [16]. In folk medicine, Allium was used to treat the rheumatic and inflammatory disorders [17, 18], gout, arthritis, psoriasis, hemorrhoid, diarrhea, stomach pain [19] and gastrointestinal disorders [13, 20].

Allium species including garlic (*Allium sativum*) and common onion (*Allium cepa*) are magnificently nutritious as well [21]. In this regard, garlic or extracts obtained from garlic are being incorporated into functional foods as natural antimicrobial agents [22]. These species are cultivated and traded due to their availability across the world and their leaves and bulbs are used raw or cooked in foods [23, 24].

Accumulating evidence recognize Allium species as a rich source of secondary metabolites such as flavonoids, alkaloids, organosulfur compounds and saponins, which possess antibacterial, antiviral, antifungal, antihelmintic, antiprotozoal and anticancer properties [14, 25–31]. Among Allium species, *Allium bakhtiaricum* is native to Iran and grows on the Zagros mountains. Besides its application in traditional medicine, this plant is used to prepare a broad range of local foods. To date, *Allium jesdianum*, a similar species to *A.bakhtiaricum* has been found to have pharmacological properties including analgesic effect [32], inhibition of platelet aggregation [33] and renal stone formation [34] as well as anticancer activity [35]. To the best of our knowledge, there is no investigation of the anticancer activity of *A.bakhtiaricum* extracts. This motivated us to explore the in vitro and in vivo anticancer activity of different extracts obtained from *A. bakhtiaricum* aerial parts.

## Methods

### Plant material and preparation of extracts

*A.bakhtiaricum* was collected from Shiraz, Iran, in the spring, authenticated by Dr.Shahin Zarre and deposited at the Herbarium of Faculty of Sciences, Tehran University, Tehran, Iran (Voucher No:45496). The aerial parts were air dried prior to being grinded into powder. 50 g of dried powder was mixed with ethanol: water (80:20) at room temperature in order to obtain total extract. In addition, 100 g of plant powder was extracted sequentially by solvents with a wide range of polarities including n-hexane, chloroform, ethyl acetate and methanol using a maceration procedure. The process was repeated 3 times with the same plant material but using fresh solvents [36–38]. The extracts were then filtered and evaporated to dryness on a rotary evaporator under reduced pressure below 40 °C. All the extracts were stored at 4 °C until used for experiments. The yield of extraction for total extract, n-hexane, chloroform, ethyl acetate and methanol fractions were as follows: 32.57, 1.63, 1.08, 0.4 and 15.25%, respectively.

### Chemicals and cell lines

MDA-MB-231 (human breast adenocarcinoma, C578), MCF-7 (human breast adenocarcinoma, C135), HT-29 (human colorectal adenocarcinoma, C466), HepG2 (liver hepatocellular carcinoma, C158), 4 T1(mouse mammary tumor, C604) and NIH3T3 (mouse embryonic fibroblasts, C156) cell lines were purchased from the cell bank of Pasture Institute of Iran (NCBI). Cells were cultured in Dulbecco’s Modified Eagle’s Medium (DMEM) medium containing 10% fetal bovine serum (FBS), 100 U/ml penicillin and 100 μg/ml streptomycin (GibcoBRL, Rockville, IN, USA) at 37 °C with 5% CO2 in a humidified atmosphere inside a CO2 incubator. All solvents used were of analytical grade and purchased from Merck (Darmstadt, Germany), and the other chemicals were obtained from Sigma-Aldrich (St Louis, MO, USA).

### In vitro cytotoxicity assay

The cytotoxic effects of total extract and fractions were assessed toward cancerous and non-cancerous cell lines by applying the MTT assay. Following seeding of the cells (MDA-MB-231, 3–7 × 10^3^; MCF-7, 4–8 × 10^3^; HT-29, 4–8 × 10^3^; HepG2, 5–9 × 10^3^; NIH3T3, 1 × 10^3^) in 96-well plates for different time exposures, samples were added at concentrations ranging from 0.002 to 0.25 mg/ml to each well and then incubated for 24, 48 and 72 h. The cultivation media without extract was used as a negative control, while DMSO (dimethyl sulfoxide) (0.5%) served as the solvent control. Afterwards, cells were subjected to MTT (3-(4,5-Dimethylthiazol-2-yl)-2,5-diphenyl tetrazolium bromide) (0.5 mg/ml in phosphate buffered saline) for 4 h at 37 °C. Following the solubilization of the formed crystal formazan in DMSO, the absorbance was measured at 545 nm. The IC_50_ values were calculated from dose-response curves at 24, 48 and 72 h exposure times.

### Flowcytometry analysis

Cell cycle phase distribution was determined by flowcytometry. Following exposure of MDA-MB-231 cells to the ¼ IC_50-72h_, ½ IC_50-72h_ and IC_50-72h_ concentrations of the chloroform and ethyl acetate fractions for 48 h, cells were collected and stained with Propidium Iodide (PI) reagent at 37 °C for 15 min in the dark. PARTEC flowcytometer (Partec GmbH, Munster, Germany) using Flowjo Software was applied for determining the DNA content [38].

### Annexin-V staining assay

In order to detect apoptosis, Annexin-V/PI assay was carried out on the cells treated with ¼ IC_50-72h_, ½ IC_50-72h_ and IC_50-72h_ concentrations of the chloroform and ethyl acetate fractions at different times. Afterwards, 100 μl Annexin-V-FLUOS (Roche Applied Science, Indianapolis, IN, USA) labeling solution was added to the suspended MDA-MB-231 cells and incubated at 37 °C. The tubes were then diluted with buffer prior to being subjected to flowcytometry [39].

### Western blot analysis

MDA-MB-231 cells treated with the chloroform extract at ¼ IC_50-72h_, ½ IC_50-72h_ and IC_50-72h_ concentrations for 48 h were lysed in lysis buffer (Tris 62.5 mM(pH 6.8), DTT 50 mM, SDS 10%, glycerol), and the extracted proteins were separated by 15% SDS-PAGE, electroblotted to polyvinylidene fluoride membrane (GE Health Care Life Sciences, Buckinghamshire, UK) and probed with primary antibody (LC3, 1:1000) (Cell Signaling Technology,Beverly, MA), followed by anti-rabbit IgG horseradish peroxidase (HRP) secondary antibody (1:8000) (Cell Signaling Technology, Beverly, MA). Protein bands were then detected by ECL (Enhanced chemiluminescence) advanced Western blotting detection kit (General Electric Health Care Life Sciences, Buckinghamshire, UK). Image J was used for data analysis to determine integrated density of bands. Protein concentration was measured using the Bradford assay [40].

### Senescence-associated-β-galactosidase (SA-β-gal) staining

MDA-MB-231 cells were treated for 2 h with high concentrations of the ethyl acetate (0.3, 0.06 mg/ml) and chloroform(0.2, 0.05 mg/ml) fractions and then the culture media were renewed. Besides, MDA-MB-231 cells were also treated with 0.002 and 0.001 mg/ml of the chloroform as well as 0.003 and 0.001 mg/ml of the ethyl acetate fractions. After washing the cells in PBS and fixing them with 2% formaldehyde, cells were incubated at 37 °C (no CO_2_) with fresh senescence associated β-Gal (SA-β-Gal) (Abcam, Cambridge, MA) stain solution [41]. Stained cells were visualized maximal in 12–16 h. β-galactosidase activity was tracked under a Nikon eclipse TS100 inverted microscope for incidence of senescence.

### Immunofluorescence microscopy of α-tubulin

MDA-MB-231 cells were seeded on 8-well glass slides(SPL Life Sciences, Korea) and treated with ¼ IC_50-72h_ concentration of the chloroform extract for 48 h. Having washed with phosphate-buffered saline, cells were fixed in glutaraldehyde (1% in PBS) at room temperature for 10 min. Afterwards, the fixed cells were washed again by PBS and permeabilized using washing buffer (0.1% Triton X-100, 1% bovine serum albumin in TBS) for 10 min. Cells were then incubated for 30 min with mouse anti-α-tubulin monoclonal antibody (1:100) (Sigma-Aldrich, USA) at room temperature followed by incubation with FITC conjugated anti-mouse IgG antibody (1:500) (Bioscience, CA) for 30 min. The nuclei were stained with propidium iodide (10 mg/ml) (Sigma–Aldrich, USA) [42, 43]. Microtubule networks were detected under a Nikon H600L fluorescence microscope.

### In vivo study

Female BALB/c mice (6–8 week old) were purchased from the National Animal Center (Pasteur Institute of Karaj) and maintained under standard conditions of 12/12-h light–dark cycle, with food and water provided ad libitum. Treatment of animals was performed in accordance with the guidelines approved by the animal ethics committee of Pasteur Institute of Iran. Mice were inoculated with 10^6^cells/50 μl of exponentially 4 T1 cells into the mammary fat pad. Following daily observation, once the tumor masses were developed, mice bearing tumor were randomly distributed into eight groups (*n* = 8). To detect the tumor suppressive role of the chloroformic and ethyl acetate fractions, mice were daily administered by intra-peritoneal injection of vehicle alone (DMSO) and three doses of the chloroformic and ethyl acetate fractions (1, 10 and 20 mg/kg) 5 days a week for 28 days. Mice received no treatment in the negative control group. During the experiments, the tumor growth was tracked every other day and the tumor volume was determined in two dimensions thrice a week using a digital caliper. The tumor volume (mm^3^) was calculated according to the formula: (length × width^2^)/2. Treated mice were daily monitored for toxicity including weight loss, discomfort and death. Following anesthesia with 60 mg/kg of sodium pentobarbital, the mouse chest was surgically opened and concurrently perfused by 0.9% saline and then tissue samples including the tumor and lungs were dissected from the animals. Samples were weighed, measured and then placed in 10% formalin for fixation and histopathological analysis.

### Histopathology of the tumors

The fixed tissues were embedded into paraffin blocks, and 5 μm sections were prepared. The tissue sections were picked up onto a glass slide and deparaffinized, rehydrated, and subjected to Hematoxylin and Eosin (H&E) (Merck, Darmstadt, Germany) staining. A Carl Zeiss AxioImager microscope and Image M1 Software (Carl Zeiss, Jena, Germany) were used to provide images of randomly chosen fields at 400x magnification.

### Statistical analysis

The data are expressed as mean ± SEM of at least triplicate determinations. Differences between the groups were evaluated by using one-way analysis of variance (ANOVA) followed by posthoc Tukey multiple comparison test using Graph Pad Prism 6 software. *P* value less than 0.05 was considered as significant.

## Results

### Cytotoxic activity of *Allium bakhtiaricum* extracts

IC_50_ values of total extract and different fractions of *A.bakhtiaricum* upon 24, 48 and 72 h of treatment toward all the tested cell lines are presented in Table 1. All the extracts caused loss of cell viability in a concentration and time dependent manner on the cell lines. Among different incubation times, a strong cytotoxic activity was revealed following 72 h of incubation of the cancerous cell lines with *A.bakhtiaricum* extracts. As shown in Table 1, the lowest IC_50_ values for the extracts were recorded as follows: total < chloroform ≤ ethyl acetate fractions at 72 h. Remarkably, of all the cell lines, MDA-MB-231 cell line was the most sensitive to the chloroformic and ethyl acetate fractions with IC_50_ values of 0.005 and 0.006 mg/ml, respectively.

**Table 1:** IC_50_ values (mg/ml) for cytotoxic activity of different extracts towards MDA-MB-231, HT-29, HepG2 and MCF-7 cells ^a^

Cell Line/Time	MDA-MB-231			HT-29			HepG2			MCF-7		
	24 h	48 h	72 h	24 h	48 h	72 h	24 h	48 h	72 h	24 h	48 h	72 h
Extracts												
Total	0.03 ± 1.12	0.02 ± 1.09	0.004 ± 1.07	0.08 ± 1.06	0.03 ± 1.06	0.01 ± 1.06	0.05 ± 1.05	0.05 ± 1.05	0.04 ± 1.05	0.05 ± 1.06	0.04 ± 1.06	0.02 ± 1.07
Hexane	> 0.25	> 0.25	0.02 ± 1.15	> 0.25	> 0.25	0.08 ± 1.10	> 0.25	> 0.25	> 0.25	> 0.25	> 0.25	0.2 ± 1.10
Chloroform	0.07 ± 1.05	0.03 ± 1.10	0.005 ± 1.12	0.04 ± 1.09	0.03 ± 1.12	0.02 ± 1.11	0.08 ± 1.07	0.06 ± 1.08	0.04 ± 1.08	0.06 ± 1.06	0.03 ± 1.08	0.02 ± 1.09
Ethyl acetate	0.07 ± 1.06	0.04 ± 1.13	0.006 ± 1.10	0.07 ± 1.09	0.05 ± 1.08	0.02 ± 1.09	0.1 ± 1.08	0.1 ± 1.07	0.07 ± 1.10	0.08 ± 1.06	0.06 ± 1.08	0.03 ± 1.06
Methanol	0.16 ± 1.03	0.05 ± 1.09	0.01 ± 1.06	0.12 ± 1.06	0.07 ± 1.07	0.04 ± 1.09	0.2 ± 1.07	0.1 ± 1.06	0.07 ± 1.07	0.09 ± 1.05	0.06 ± 1.06	0.05 ± 1.07

^a^Values were determined from at least three independent experiments each performed in triplicate and expressed as mean ± SE

In order to explore cell viability of the most effective fractions in a normal cell line, the MTT assay was also performed on NIH3T3 (mouse embryo fibroblast) after 72 h of treatment. Our results indicated that the IC_50_ values of the chloroformic and ethyl acetate fractions (0.05 and 0.08 mg/ml) were higher against NIH3T3 cells than those obtained from these fractions on cancer cell lines.

### Cell cycle arrest induced by the chloroformic and ethyl acetate fractions in MDA-MB-231 cells

Since progression of the cells through the various phases of the cell cycle results in cell proliferation [44], we next evaluated the effects of both chloroform and ethyl acetate fractions, as the most cytotoxic extracts, on cell cycle distribution of MDA-MB-231 cell line using flowcytometry. To do this, cultured MDA-MB-231 cells were exposed to the IC_50-72h_, ½ IC_50-72h_ and ¼IC_50-72h_ concentrations of the chloroform and ethyl acetate fractions for 48 h, stained with PI and analyzed by flowcytometry. Our findings revealed that MDA-MB-231cells treated with ¼ and ½IC_50-72h_ concentrations of the chloroformic and ethyl acetate fractions caused a statistically significant rise in the percentage of cell population in G2 phase after 48 h (Table 2). Thus, it appears that these fractions are able to cause cell cytotoxicity by modulating the cell cycle profile and inducing G2/M phase arrest.

**Table 2:** Effect of chloroform and ethyl acetate fractions at different concentrations on MDA-MB-231 cell cycle progression^a^

Concentration (mg/ml)	Sub-G1	G1	S	G2
Chloroform extract				
(0.005)	16.9 ± 1.5	43.87 ± 1.86	33.85 ± 1.45	12.17 ± 1.01
(0.0025)	14.95 ± 1.95	43.20 ± 3.60	35.63 ± 0.66	13.43 ± 1.2*
(0.0012)	10.95 ± 0.15	47.90 ± 2.61	30.98 ± 1.51	20.90 ± 0.5****
Control/Vehicle	12.23 ± 0.89	46.58 ± 0.91	32.85 ± 0.92	9.58 ± 0.57
Ethyl acetate extract				
(0.006)	20.30 ± 0.3*	48.30 ± 1.8	30.87 ± 1.35	9.21 ± 1.15
(0.003)	7.22 ± 1.01	47.60 ± 3.2	29.7 ± 2.39	14.01 ± 0.63**
(0.0015)	7.65 ± 1.33	46.83 ± 1.2	28.53 ± 1.73	16.75 ± 0.45***

^a^The data presented are the mean ± SE of three independent experiments. *p < 0.05, **p < 0.01,

***p < 0.001,****p < 0.0001 relative to control-vehicle

### Apoptosis evaluation by Annexin-V/PI staining

To ascertain whether cytotoxic effect of the chloroform or ethyl acetate fraction was due to apoptosis induction, MDA-MB-231 cells were labeled using annexinV/PI and analyzed by flowcytometry. Following treatment with IC_50-72h_, ½ and ¼ IC_50-72h_ concentrations of chloroformic and ethyl acetate fractions, the percentage of viable cells were significantly declined after 72, 48, 24 and 12 h of incubation; however no significant increase was observed in the number of apoptotic cells. Of note, an upward trend was observed in the population of necrotic cells with increasing concentration of the fractions from ¼ IC_50-72h_ to IC_50-72h_ values. Also, apoptotic change was not detected after 6 h of exposure of the cells to the fractions (Additional file 1). As a whole, our results implied that ethyl acetate and chloroform extracts possess a non-apoptotic anti-cancer activity.

### Monitoring autophagy after treatment of MDA-MB-231 cells with the fractions

**Fig. 1:**
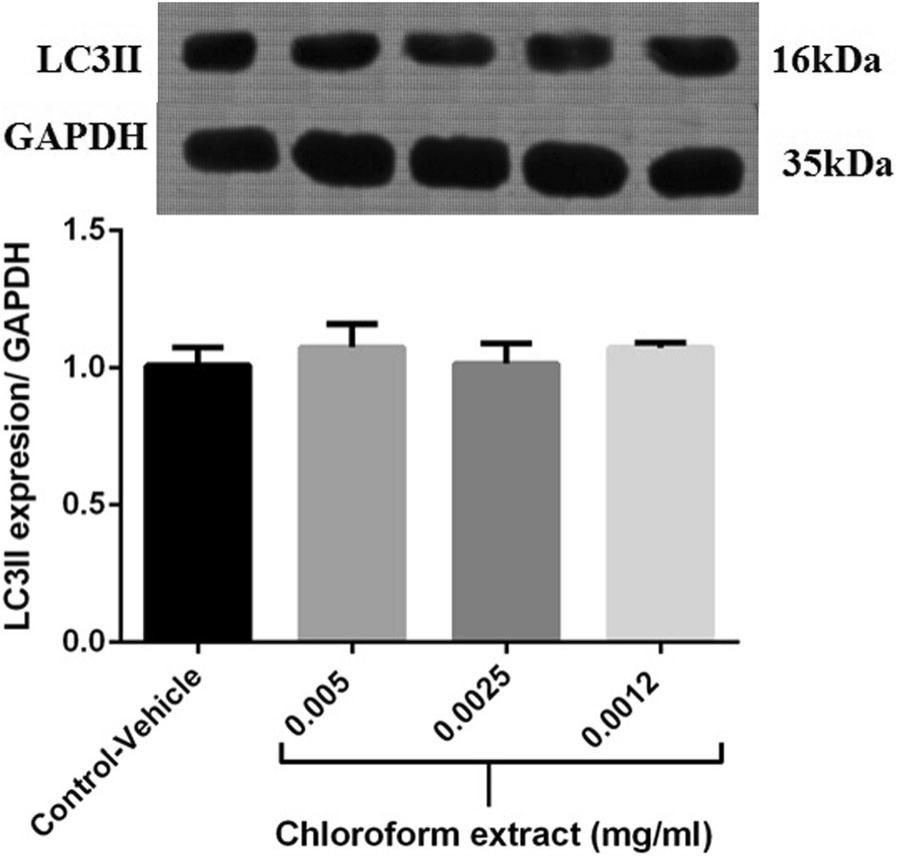
Western blot analysis of LC3-II expression in MDA-MB-231 cells treated with the chloroform extract at 0.005, 0.0025 and 0.0012 mg/ml for 48 h. GAPDH was used as a loading control. Protein band intensities were quantified by Image J. Data are the mean ± SE of three separate experiments

To determine whether cell death was resulted from autophagy activation, LC3-II accumulation in MDA-MB-231 cells, treated for 48 h with different concentrations of the chloroformic extract, was assessed by Western blotting. A hallmark of autophagy activation is the change of the cytosolic form of LC3 (LC3-I) to its lipidized form (LC3-II) [45]. Hence, we compared the LC3 expression in the chloroformic fraction-treated and untreated MDA-MB-231 cells. As our findings show, upon treatment of MDA-MB-231 cells with the chloroformic fraction, no significant change is observed in the LC3-II expression (Fig. 1.). Regarding Western blotting, the incidence of controlled autophagy in the untreated cell was shown by the presence of LC3-II.

### Senescence investigation in the chloroformic and ethyl acetate extracts-treated MDA-MB-231 cells

**Fig. 2:**
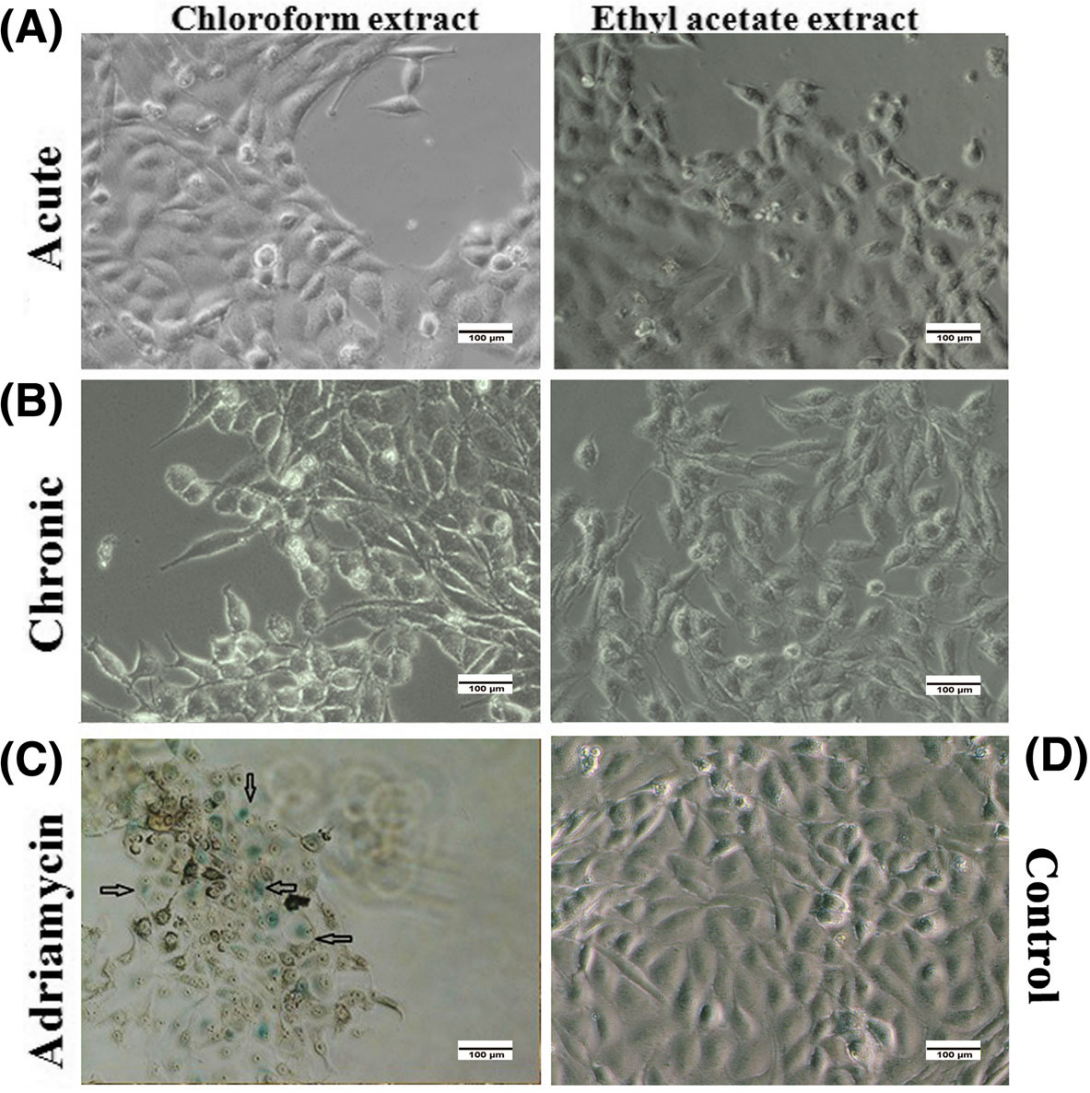
Acute and chronic treatment with the chloroformic and ethyl acetate fractions did not induce cell senescence. MDA-MB231 cells were treated with (**a**) high concentrations of chloroformic (0.2, 0.05 mg/ml) and ethyl acetate (0.3, 0.06 mg/ml) fractions and (**b**) low concentrations of chloroformic (0.001, 0.002 mg/ml) and ethyl acetate (0.001, 0.003 mg/ml) fractions for 10 days and analyzed for the senescence-associated β-galactosidase activity. (**c**) Arrows show blue cells with a typical senescent flattened and enlarged morphology in MCF-7 cells treated with 1 μM adriamycin as a positive control and (**d**) untreated MDA-MB231 cells were considered as a negative control. The image shown is representative of at least three independent experiments

It has been proposed that plant derived compounds may exert their anticancer activity via senescence mechanism due to the presence of alkaloids [46]. In addition, cell growth arrest is likely through the senescence induction [47]. Considering our findings, which indicated a cell cycle arrest following the treatment with the extracts and knowing that Allium possess a wide variety of natural compounds such as alkaloids [48, 49], we sought to explore whether a low dose and chronic treatment of the chloroform and ethyl acetate fractions could prevent the growth of breast cancer cells by inducing the premature senescence. Finding β-galactosidase positive cells implies an increased lysosomal mass and it is commonly considered as a well-established senescence marker [41]. We noticed that MDA-MB-231 cells treated with the chloroformic and ethyl acetate fractions showed no detectable SA-β-gal activity as illustrated in Fig. 2a, b. In contrast, the SA-β-gal positive cells, treated with 1 μM of Adriamycin, exhibited a blue color with a flattened and enlarged morphology, which are indicatives of senescence features (Fig. 2c) [50].

### Disarrangement of microtubules induced by the chloroform fraction

**Fig. 3:**
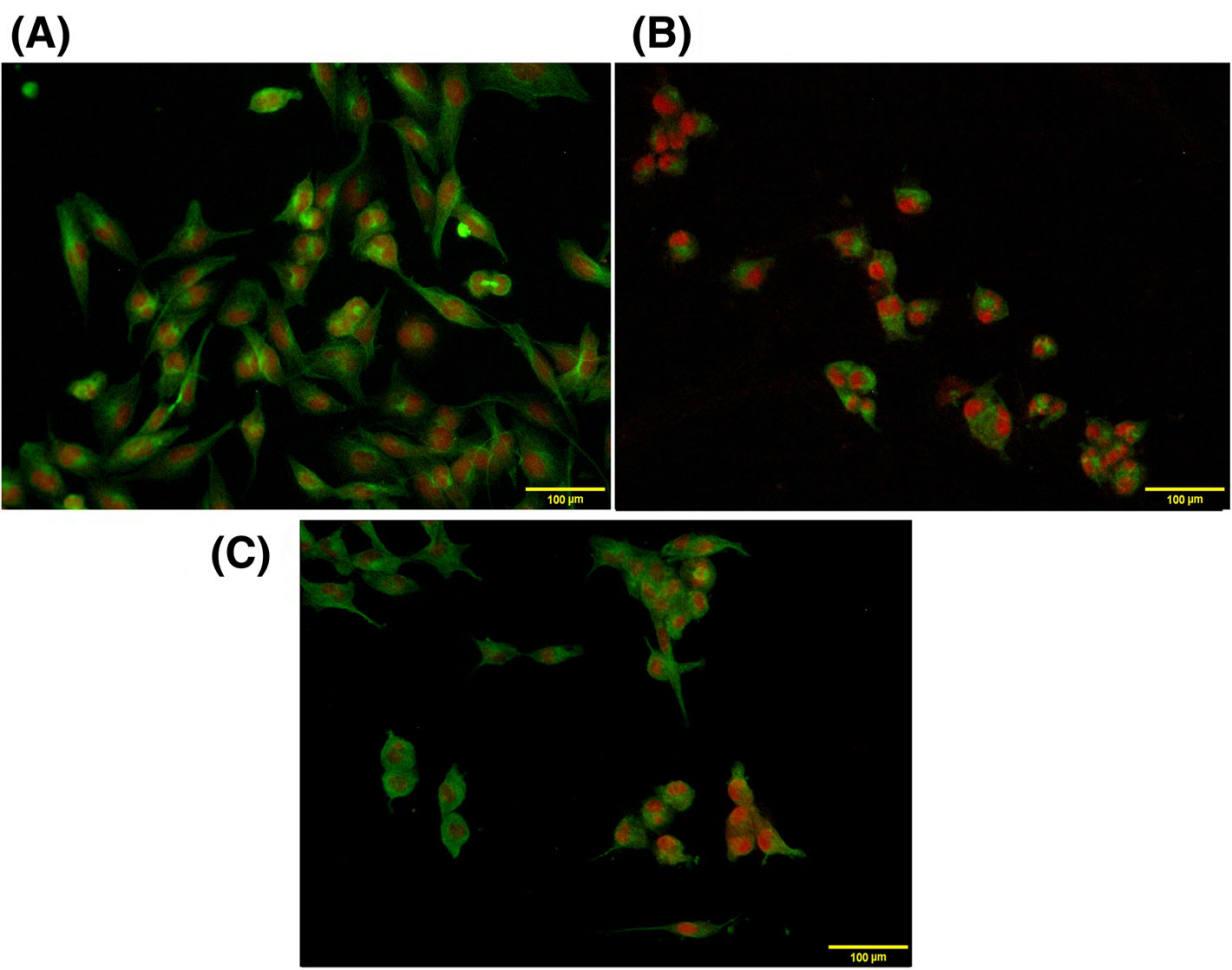
Effect of the chloroformic extract on the organization of cellular microtubule network. The microtubule network (green) and the Nuclei (red) are shown in (**a**) MDA-MB-231 cells; MDA-MB-231 cells were treated with (**b**) 4 μM of paclitaxel, and (**c**) ¼ IC_50-72h_of the chloroform extract after a 48 h incubation. The cellular microtubules were observed by Nikon H600L fluorescence microscope (original magnification 400x)

Because microtubule network perturbation can account for the subsequent mitotic arrest [43], herein, we examined whether the chloroform fraction treatment affected the cellular microtubule network. To do this, MDA-MB-231 cells were treated with 4 μM paclitaxel and ¼ IC_50-72h_ concentration of the chloroform extract, separately. Following 48 h of incubation, the microtubule network was observed by immunocytochemistry (Fig. 3). A normal arrangement and organization of microtubule network was visualized in the control cells (Fig. 3a), whereas paclitaxel enhanced microtubule density and caused long thick microtubule bundles to appear around the nucleus (Fig. 3b). Treating cells with the chloroformic fraction resulted in similar changes as those of paclitaxel-treated cells, demonstrating microtubule network as a possible intracellular target for components of the chloroform fraction. In order to quantify these findings, we calculated the percentage of cells bearing microtubule disarrangement and compared it to the paclitaxel-treated cells. The results were as follows: 96.95 ± 3.04% for paclitaxel- and 83.33 ± 4.16% for the chloroformic fraction-treated cells. Our outputs indicated a significant change in the microtubule network of the cells treated with the chloroformic extract.

### Chloroformic and ethyl acetate fractions suppressed primary tumor growth

**Fig. 4:**
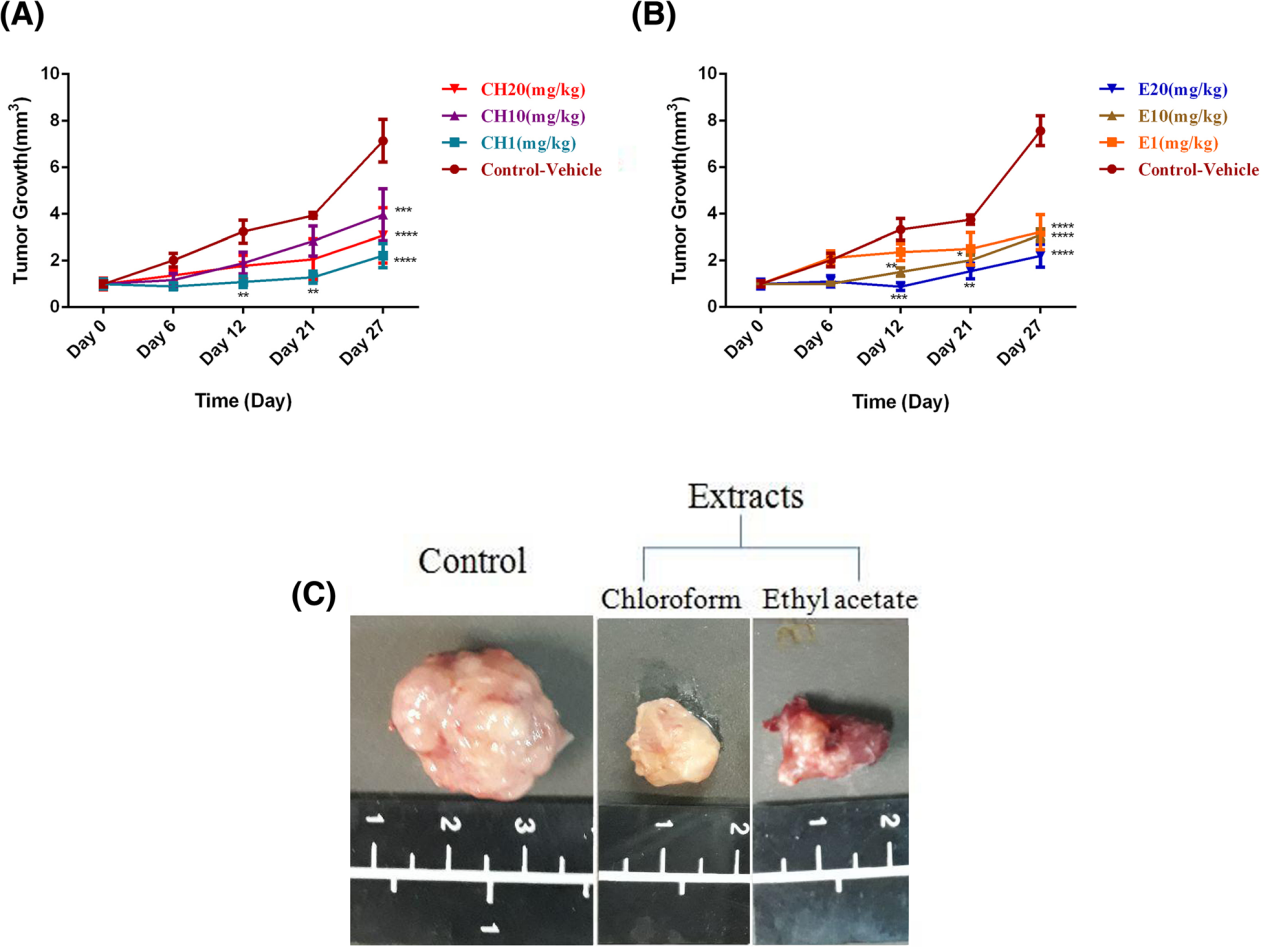
Different doses (1, 10, 20 mg/kg) of (**a**) chloroform and (**b**) ethyl acetate fractions affected primary tumor growth after 4 weeks of daily treatment. (**c**) Images of tumors harvested from control and the treated mice (1 mg/kg). Data are expressed as mean ± SEM, n = 8 mice per group

To verify the antitumor activity of the chloroform and ethyl acetate fractions in vivo, BALB/c mice were subcutaneously (s.c.) injected with 4 T1 murine tumor cells. Following tumor development on day 7, the mice were distributed in 8 groups and treated with either the vehicle or the chloroformic and ethyl acetate fractions (1, 10, 20 mg/kg/day) for 21 days. As illustrated in Fig. 4a.,i.p. administration of 1 mg/kg/day of the chloroform extract led to a significant tumor growth suppression from the second week (*p* < 0.01) to the last week (*p* < 0.0001) of the treatment compared to the vehicle-control group. However, tumor volume reduction was observed after 4 weeks of treatment with 10 and 20 mg/kg/day of the chloroform extract. On the other hand, i.p. administration of 1 mg/kg/day of the ethyl acetate fraction was unable to significantly inhibit tumor growth within the 3 weeks of injection, and a remarkable potency was only observed following 4 weeks of treatment (Fig. 4b). With an increase in the dose of ethyl acetate fraction from 1 to 20 mg/kg/day, tumor growth began to regress significantly compared to the control animals from week 2 to 4 of the treatment. The maximum effect in terms of tumor size was seen for the chloroform fraction following 4 weeks of treatment at 1 mg/kg/day. Consistent with these data, tumor weight was also different among the control and the treated groups. The tumor weight was reduced from 2.02 ± 0.27 g in the control group to 1.01 ± 0.34 g and 0.98 ± 0.11 g in the chloroform and ethyl acetate treated groups, respectively. Also, according to Fig. 4c, the size of the tumor in the control group was larger than that of the treated mice. Remarkably, no weight loss was detected either in the control-vehicle or the treated groups after 28 days.

### Effect of the chloroform and ethyl acetate fractions on the mammary tumor tissues and metastasis to the lung

**Fig. 5:**
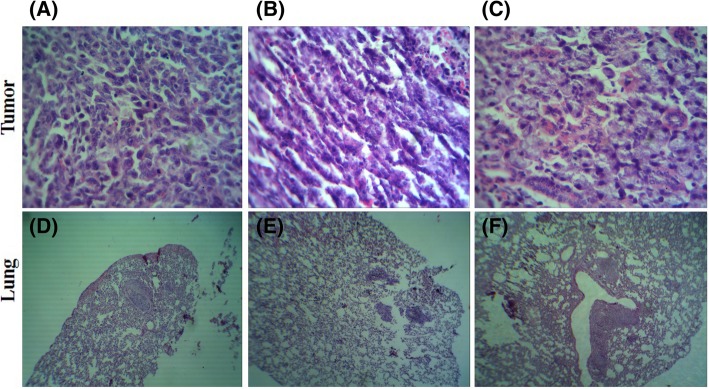
Effect of the chloroformic and ethyl acetate fractions at 1 mg/kg/day on solid tumors in Balb/c mice injected with 4 T1 cells. The mice were killed 32 days after cell injection. (**a**-**c**) tumor and (**d**-**f**) lung sections following H&E staining (original magnification 400x and 100x)

In the mice bearing 4 T1, the tumor tissues displayed malignant cells with the features including loss of polarity and a number of typical mitotic figures (Fig. 5a). These types of cells were nominated as an invasive lineage representing a suitable model to evaluate the potency of anticancer drugs due to its similarities with metastatic human breast cancer [51]. In the groups treated with 1 mg/kg/day of the chloroform or ethyl acetate fractions, a number of tumor cells in the process of necrosis were observed (Fig. 5b, c); however, tumors dissected from mice treated with the chloroform fraction presented a reduced number of proliferative cells than those from the ethyl acetate fraction at the same dose. These findings are in line with our tumor volume determinations, further corroborating a greater efficiency for the chloroformic fraction in suppressing tumor growth.

The findings of histopathology also exhibited less foci of metastasis in the lungs of the animals treated with chloroform and ethyl acetate fractions compared to the vehicle-treated animals, indicating an anti-invasive property of the fractions (Fig. 5d-f). Besides, a difference was observed between the metastatic lung foci of the chloroform and ethyl acetate treated groups, with the chloroform treated lung tissues harboring less nodules than those of the ethyl acetate-treated group.

## Discussion

Natural phytochemicals are well-known for having beneficial impacts in treatment of a broad range of illnesses. Total extracts obtained from medicinal plants contain a number of ingredients with more effective therapeutic properties and fewer side effects than a single natural compound alone [52]. It is noteworthy to mention that an extract may contain a wide variety of compounds affecting different pathways involved in cell death [53], a property that may be advantageous in treating diseases involving multiple mechanisms such as cancer [54]. Allium vegetables have been traditionally used for centuries in Asian, American and European medicine to alleviate diseases [55], but recently, a number of studies have been carried out on anti-cancer activity of Allium species [14, 25, 26, 56]. Allium species have numerous uses in folk medicine in Iran [34], however no studies have been reported on the anticancer activity of *Allium bakhtiaricum*. In the present study, we focused on evaluating the influence of *A.bakhtiaricum* extracts on the proliferation of different cancer cells along with their possible underlying mechanisms. In addition, we further verified the antitumor effect of the most potent extract in an in vivo model.

The current study revealed for the first time that *A.bakhtiaricum* extract is capable of inhibiting the growth of breast cancer cells. In this regard, *A.bakhtiaricum* total extract and different fractions significantly diminished cell viability in a concentration- and time-dependent manner. According to the obtained IC_50_ values, efficacy of the extracts were in the order of total > chloroform > ethyl acetate > methanol extracts at three different times (24, 48 and 72 h). Notably, among the different tested cell lines, MDA-MB-231 cells were the most sensitive in response to all the extracts. Our findings also revealed that the chloroformic and ethyl acetate fractions had minimal cytotoxic effect on normal cells with IC_50_ values of 0.05 and 0.08 mg/ml, respectively, indicating a selective inhibitory impact on cancer cells with no effect on normal cells. Hence, these two fractions could be considered as potential chemotherapeutics in cancer therapy.

In order to detect the type of cell death operating in cells treated with chloroform and ethyl acetate treatments as the most effective extracts, different cell death mechanisms were investigated. Our AnnexinV/PI test displayed a minimal cell apoptosis in the late stage upon exposure of MDA-MB-231 cells to ½IC_50-72h_ concentration of the chloroform extract after 24 h. On the other hand, a large percentage of cells appeared in the necrotic stage at 24 h and even at less upon exposure of breast cancer cells to both fractions at ½IC_50-72h_ and ¼ IC_50-72h_ concentrations. Despite being incapable of inducing major apoptosis in cancer cells, the two fractions, however, caused cell death through induction of cell cycle arrest at G2/M phase at ¼ and ½ IC_50-72h_ concentrations in MDA-MB-231 cells after 48 h of treatment. These observations suggest a preventive effect for the extracts against cancerous cells entering into mitosis phase, a property associated with the mechanism of the action of most anticancer drugs [57]. We next were interested in whether senescence can be a causative trigger for cell cycle arrest, thus, we examined the treated cells by SA-β-gal staining. Our results showed that long-term incubation with low concentrations (0.001 and 0.002 mg/ml) of the chloroformic as well as ethyl acetate (0.001 and 0.003 mg/ml) fractions induced no senescence-like cell growth arrest. Similarly, no sign of senescence was detected following acute treatment of MDA-MB-231 cells with the chloroformic fraction at 0.2 and 0.05 mg/ml along with the ethyl acetate extract at 0.3 and 0.06 mg/ml.

On the other hand, multiple lines of evidence indicate that suppression of apoptosis can result in autophagy activation [58]. This assumption encouraged us to evaluate autophagy in MDA-MB-231 cells treated with the extracts. As demonstrated by our results, the chloroformic fraction did not affect the expression level of LC3-II, the autophagic marker, at concentrations in which cell cycle arrest occurred at 48 h. These results are consistent with our findings on senescence, supporting the notion that cellular senescence and autophagy are closely linked events [40]. Therefore, we observed neither senescence nor autophagy upon treatment of breast cancer cells with the extracts. To fully elucidate the operating mechanism(s) of cell death would, therefore, require further investigations.

An interesting result in the current study was the similarity between the effects of the chloroformic fraction on tubulin polymerization at ¼ IC_50-72h_ concentration to that of paclitaxel, where thick bundles of microtubules appeared around the nucleus. These findings suggest alteration in microtubule dynamics as a probable mechanism through which the chloroform fraction may act, leading to cell cycle arrest within G2/M, a role associated with naturally occurring antimitotic agents [59]. It will be of interest to design further experiments to ascertain this hypothesis.

In order to verify the anticancer potential of the two extracts, a syngenic mice model using 4T1 cell line was selected due to its high reproducibility and suitability for testing the efficiency of chemotherapeutic agents used for treating breast cancer, particularly metastatic breast cancer [60]. We found that the daily administration of the chloroformic and ethyl acetate fractions were able to suppress tumor growth in the murine tumor models at 1, 10 and 20 mg/kg based on the regression of tumor volumes following 4 weeks of treatment, which are in line with the in vitro results. Importantly, the chloroform fraction showed more efficiency in tumor volume reduction at different doses within 4 weeks of treatment compared to the ethyl acetate fraction. Moreover, our findings corroborate that 1 mg/kg of both extracts is the minimum effective dose to reduce tumor volume. Furthermore, after a significant drop in tumor volume upon treatment with both fractions in the first week, the tumor size remained constant within the following 3 weeks, demonstrating a rapid onset of action with a good penetration into the tumor. These results may be related to the presence of natural compounds including organosulfurs, saponins, terpenoids, alkaloids and flavonoids in the extracts of Allium species, which make them suitable candidates in fighting cancer [5, 14, 15, 53]. Notably, the anticancer property speculated for most allium species due to the presence of flavonoids, is through promoting apoptosis [61], thus, we assume that the non-apoptotic cell death occurred in the current study is most likely associated with the presence of other natural bioactives.

Consistently, H&E staining of the tumors also revealed a reduction in the number of actively dividing cells, a hallmark of cancer cells, following the injection of the two fractions at 1 mg/kg/day, compared to the control-vehicle; however, more cells in the process of necrosis were observed in the group administered with the chloroform fraction than with the ethyl acetate group. In addition, the survival rates of mice were indicative of a good tolerance towards the treatments, reinforcing the idea that the total death occurred as a result of the tumor rather than the toxicity of the extracts. The inhibitory effect of both fractions on the spreading of the 4 T1 breast cancer cells to a secondary site, i.e., lung, was also diminished in the treated groups, as recognized by a reduction in the number of metastatic nodules. In agreement with our previous data, the development of lung metastasis was lower in the chloroform-treated mice bearing 4 T1 cells than in the ethyl acetate-treated group.

## Conclusion

For the first time, the findings of the current study demonstrated that the chloroform extract of *A.bakhtiaricum* potently suppressed the tumor growth in vitro and in vivo, a property which may be attributed to the presence of bioactive compounds in this plant with anti-breast cancer potential. Experiments to further identify these active ingredients as well as the mechanisms of their anticancer activity are presently ongoing in our laboratory.

## Additional files

Additional file 1: **Table S1.** Percentage of MDA-MB-231 cells in each state after treatment with the fractions at 72 h. **Table S2.** Percentage of MDA-MB-231 cells in each state after treatment with the fractions at 48 h. **Table S3.** Percentage of MDA-MB-231 cells in each state after treatment with the fractions at 24 h. **Table S4.** Percentage of MDA-MB-231 cells in each state after treatment with the fractions at 12 h. **Table S5.** Percentage of MDA-MB-231 cells in each state after treatment with the fractions at 6 h. (DOCX 28 kb)

## Abbreviation

A.bakhtiaricum: Allium bakhtiaricum
A.cepa: *Allium cepa*
A.sativum: *Allium sativum*
DMEM: Dulbecco’s Modified Eagle’s Medium
DMSO: Dimethyl Sulfoxide
ECL: Enhanced Chemiluminescence
FBS: Fetal Bovine Serum
MTT: 3-(4,5-Dimethylthiazol-2-yl)-2,5-diphenyltetrazolium bromide
SA-β-gal: Senescence Associated β-galactosidase
