# Supplementary material for: Potent antitumor property of Allium bakhtiaricum extracts
Source: BMC Complement Altern Med. 2019 Jun 4;19:116. doi: 10.1186/s12906-019-2522-8 (PMC6549325; doi:10.1186/s12906-019-2522-8)
Supplement: Supplementary file 1 — Table S1. Percentage of MDA-MB-231 cells in each state after treatment with the fractions at 72 h. Table S2. Percentage of MDA-MB-231 cells in each state after treatment with the fractions at 48 h. Table S3. Percentage of MDA-MB-231 cells in each state after treatment with the fractions at 24 h. Table S4. Percentage of MDA-MB-231 cells in each state after treatment with the fractions at 12 h. Table S5. Percentage of MDA-MB-231 cells in each state after treatment with the fractions at 6 h. (DOCX 28 kb) [file 12906_2019_2522_MOESM1_ESM.docx]

Additional file 1: **Table S1.**Percentage of MDA-MB-231 cells in each state after treatment with the fractionsat

72h.^a^

| **Extract(mg/ml)** | **Vital cells**  **(%) An–/PI–** | **Early apoptosis (%) An+/PI-** | **Late apoptosis (%) An+/PI+** | **Necrosis (%) An-/PI+** |
| --- | --- | --- | --- | --- |
| **Chloroform** |  | | | |
| **(0.005)** | 0.81±0.41^****^ | 0.03±0.02 | 0.67±0.14 | 98.68±0.62^****^ |
| **Ethylacetate** |  | | | |
| **(0.006)** | 2.19±0.31^****^ | 0.06±0.02 | 1.97±0.12 | 94.53±1.13^****^ |
| **Control/Vehicle** | 88.51±3.22 | 0.03±0.01 | 2.42±0.46 | 8.66±2.17 |

^a^The data presented are the mean±SE of three independent experiments. *****p* < 0.0001 relative to vehicle-control

Additional file 1: **Table S2.**Percentage of MDA-MB-231 cells in each state after treatment with the fractions at 48h.^a^

| **Extract(mg/ml)** | **Vital cells**  **(%) An–/PI–** | | **Early apoptosis (%) An+/PI-** | **Late apoptosis (%) An+/PI+** | **Necrosis**  **(%) An-/PI+** |
| --- | --- | --- | --- | --- | --- |
| **Chloroform** |  | | | | |
| **(0.0025)** | 27.36±0.17^****^ | | 0.04±0.00 | 1.00±0.17 | 72.01±0.37^****^ |
| **(0.0012)** | 66.00±3.06^****^ | | 0.03±0.00 | 0.68±0.48 | 32.28±1.58^***^ |
| **Ethyl acetate** |  | | | | |
| **(0.003)** | 25.52±7.32^****^ | | 0.38±0.01 | 0.31±0.08 | 57.83±9.10^****^ |
| **(0.0015)** | 87.32±2.38 | | 0.35±0.05 | 0.43±0.01 | 9.36±0.13 |
| **Control/Vehicle** | | 93.50±0.80 | 0.15±0.06 | 1.3±0.32 | 5.55±1.00 |

^a^The data presented are the mean±SE of three independent experiments.****p* < 0.001,*****p* < 0.0001 relative to vehicle-control

Additional file 1: **Table S3.**Percentage of MDA-MB-231 cells in each state after treatment with the fractions at 24h.^a^

| **Extract(mg/ml)** | **Vital cells**  **(%) An–/PI–** | **Early apoptosis (%) An+/PI-** | **Late apoptosis (%) An+/PI+** | **Necrosis (%) An-/PI+** |
| --- | --- | --- | --- | --- |
| **Chloroform** |  | | | |
| **(0.0025)** | 31.48±5.09^****^ | 2.14±0.68 | 5.51±0.09^*^ | 56.93±1.82**** |
| **(0.0012)** | 64.24±0.51^**^ | 1.23±1.06 | 1.25±0.92 | 18.03±0.55** |
| **Ethyl acetate** |  | | | |
| **(0.003)** | 37.99±0.72^****^ | 1.44±0.93 | 3.84±1.66 | 56.15±3.22^***^ |
| **(0.0015)** | 83.73±1.76^**^ | 2.75±0.55 | 2.23±0.17 | 11.29±1.04 |
| **Control/Vehicle** | 97.16±1.08 | 0.34±0.16 | 0.65±0.23 | 0.78±0.03 |

^a^The data presented are the mean±SE of three independent experiments. **p* < 0.05,***p* < 0.01,****p* < 0.001,*****p* < 0.0001 relative to vehicle-control

Additional file 1: **Table S4.**Percentage of MDA-MB-231 cells in each state after treatment with the fractions at 12h.^a^

| **Extract(mg/ml)** | **Vital cells**  **(%) An–/PI–** | **Early apoptosis (%) An+/PI-** | **Late apoptosis (%) An+/PI+** | **Necrosis (%) An-/PI+** |
| --- | --- | --- | --- | --- |
| **Chloroform** |  | | | |
| **(0.0012)** | 93.05±0.00^***^ | 0.00±0.00 | 0.03±0.00 | 6.92±0.12^***^ |
| **Ethyl acetate** |  | | | |
| **(0.0015)** | 96.99±0.52 | 0.00±0.00 | 0.09±0.01* | 2.43±0.21 |
| **Control/Vehicle** | 98.31±0.28 | 0.00±0.00 | 0.02±0.00 | 1.43±0.26 |

^a^The data presented are the mean±SE of three independent experiments. **p* < 0.05,****p* < 0.001 relative to vehicle-control

Additional file 1: **Table S5.**Percentage of MDA-MB-231 cells in each state after treatment with the fractions at 6h.^a^

| **Extract(mg/ml)** | **Vital cells**  **(%) An–/PI–** | **Early apoptosis (%) An+/PI-** | **Late apoptosis (%) An+/PI+** | **Necrosis (%) An-/PI+** |
| --- | --- | --- | --- | --- |
| **Chloroform** |  | | | |
| **(0.0012)** | 91.59±2.11 | 2.37±1.14 | 2.35±0.26 | 2.16±0.07^****^ |
| **Ethyl acetate** |  | | | |
| **(0.0015)** | 94.82±0.65 | 1.00±0.02 | 1.90±0.03 | 1.43±0.07^****^ |
| **Control/Vehicle** | 94.39±0.98 | 1.16±0.00 | 1.15±0.11 | 0.25±0.01 |

^a^The data presented are the mean±SE of three independent experiments, *****p* < 0.0001 relative to vehicle-control.
